# Impact of race/ethnicity and area deprivation index on Alzheimer’s disease and related disorders biomarkers in the POINTER Cohort

**DOI:** 10.1002/alz.089954

**Published:** 2025-01-09

**Authors:** Jon B. Toledo, Belen Pascual, Susan M. Landau, Theresa M. Harrison, Quentin Finn, Yitbarek N Demesie, Melissa Yu, Charles Decarli, Prashanthi Vemuri, Laura D Baker, Laura Lovato, Heather M Snyder, Joseph C. Masdeu, Valory Pavlik

**Affiliations:** ^1^ Houston Methodist Research Institute, Houston, TX USA; ^2^ University of California, Berkeley, Berkeley, CA USA; ^3^ Wake Forest University School of Public Health, Winston‐Salem, NC USA; ^4^ Baylor College of Medicine, Houston, TX USA; ^5^ University of California, Davis School of Medicine, Sacramento, CA USA; ^6^ Mayo Clinic, Rochester, MN USA; ^7^ Wake Forest School of Medicine, Winston‐Salem, NC USA; ^8^ Alzheimer's Association, Chicago, IL USA

## Abstract

**Background:**

There is limited information on biomarker‐defined Alzheimer’s disease (AD) pathology in community‐recruited individuals of diverse racial and ethnic groups. Here, we assessed the association of race/ethnicity with baseline biomarkers and cognitive measures and hypothesized a lower impact of AD pathology in non‐Hispanic White (nHW) participants in the U.S. POINTER study clinical trial, which evaluates the benefits of a multidomain lifestyle intervention on cognition of community‐recruited at‐risk elderly individuals.

**Method:**

We included 879 participants from the U.S. POINTER study enrolled in the imaging substudy (∼50% of participants) who self‐identified as nHW (n=644), African American/Black (AA/B, n=132), Asian (n=29), and Hispanic (n=74). Outcomes measures included global Aβ pathology (18F‐florbetaben), temporal meta‐ROI tau (18F‐MK‐6240), MRI‐measured white matter hyperintensity volume (WMH) volumes, global clinical dementia rating (gCDR), and global, executive, and memory standardized cognitive scores. The nHW participants were selected as reference. Power transformations normalized skewed distributions. Multivariable linear regressions include age, sex, education, Framingham risk score, and area deprivation index (ADI) as covariates.

**Result:**

AA/B harbored lower WMHs, and meta‐temporal tau ROI values. In the full model, 0.5 gCDR participants had higher centiloid values, were more likely to be AA/B and Hispanic, and had a greater burden of WMHs. In addition, higher centiloid values were associated with a 0.5 gCDR in all groups except for the AA/B participants. Hispanic, AA/B, and Asian participants had lower cognitive scores than the nHW group. A higher ADI score was associated with lower global and executive cognitive scores but not with WMH volume or Aβ centiloid score.

**Conclusion:**

The analyses of the community‐based U.S. POINTER indicate that AA/B individuals present differences in AD and related dementia biomarker values. nHW had higher fully adjusted cognitive scores. These results indicate a significant heterogeneity in the cognitive testing performance and biomarker values of individuals at risk for dementia in the community.

